# Characterization of genes required for the pathogenicity of *Pectobacterium carotovorum* subsp. *carotovorum* Pcc21 in Chinese cabbage

**DOI:** 10.1099/mic.0.067280-0

**Published:** 2013-07

**Authors:** Dong Hwan Lee, Jeong-A Lim, Juneok Lee, Eunjung Roh, Kyusuk Jung, Minseon Choi, Changsik Oh, Sangryeol Ryu, Jongchul Yun, Sunggi Heu

**Affiliations:** 1 Division of Microbial Safety, National Academy of Agricultural Science, Rural Development Administration, Suwon 441-707, Republic of Korea; 2 Department of Horticultural Biotechnology and Institute of Life Science & Resources, Kyung Hee University, Yongin 441-701, Republic of Korea; 3 Department of Agricultural Biotechnology, Center for Agricultural Biomaterials, Research Institute for Agriculture and Life Sciences, Seoul National University, Seoul 151-921, Republic of Korea

## Abstract

*Pectobacterium carotovorum* subsp. *carotovorum* is a well-known plant pathogen that causes severe soft rot disease in various crops, resulting in considerable economic loss. To identify pathogenicity-related factors, Chinese cabbage was inoculated with 5314 transposon mutants of *P. carotovorum* subsp. *carotovorum* Pcc21 derived using Tn5 transposon mutagenesis. A total of 35 reduced-virulence or avirulent mutants were isolated, and 14 loci were identified. The 14 loci could be functionally grouped into nutrient utilization (*pyrD*, *purH*, *purD*, *leuA* and *serB*), production of plant cell-wall-degrading enzymes (PCWDEs) (*expI*, *expR* and PCC21_023220), motility (*flgA, fliA* and *flhB*), biofilm formation (*expI*, *expR* and *qseC*), susceptibility to antibacterial plant chemicals (*tolC*) and unknown function (ECA2640). Among the 14 genes identified, *qseC*, *tolC* and PCC21_023220 are novel pathogenicity factors of *P. carotovorum* subsp. *carotovorum* involved in biofilm formation, phytochemical resistance and PCWDE production, respectively.

## Introduction

*Pectobacterium carotovorum* subsp. *carotovorum* causes soft rot disease in cabbage, potato, onion, radish and other crops during cultivation, transportation and storage, resulting in considerable economic damage. It exists on plant surfaces and in soil, and may enter the host through wound sites or natural openings. After successfully invading a plant, it resides in the intercellular space or vascular tissue, where it produces plant cell-wall-degrading enzymes (PCWDEs) in a disease-promoting environment (Perombelon, 2002; Toth *et al.*, 2003).

Extracellular maceration enzymes, such as polygalacturonase (Peh), pectate lyase (Pel), cellulase (Cel), xylanase and protease (Prt), are important pathogenic factors of soft rot. During pathogen infection, these enzymes degrade plant structures composed of pectin, cellulose and hemicelluloses fibres, leading to plant cell necrosis and tissue maceration (Abbott & Boraston, 2008). A number of regulatory factors that control these enzymes are also important in soft rot pathogenesis. *P. carotovorum* subsp. *carotovorum* uses a cell-density-dependent cell-to-cell communication system called quorum sensing (QS) to regulate many genes and pathogenesis. In the QS system of Gram-negative bacteria, ExpI/ExpR is responsible for the synthesis of acylhomoserine lactone (AHL), which regulates the production of extracellular enzymes (Henke & Bassler, 2004; Nasser *et al.*, 1998; Pirhonen *et al.*, 1993; Whitehead *et al.*, 2002). Additionally, *flhD*, *gacA* and *hexA* participate in the regulatory pathway for PCWDE production (Cui *et al.*, 2008). Several more genes, including *cysQ*, *hel*, *yheL* and *trkA*, are reported to be involved in the production/secretion of PCWDE (Laasik *et al.*, 2005).

In addition to the above-mentioned factors, there are further requirements for *P*. *carotovorum* subsp. *carotovorum* survival in a host. Motility and nutrient uptake are important for bacterial pathogenicity in host plants. Pirhonen *et al.* (1991) reported that transposon mutants of *Erwinia carotovora* subsp. *carotovora* that were impaired in the motility-related genes had reduced virulence. Components of the flagella export apparatus are evolutionarily related to the type III secretion system (Aizawa, 2001) used for the transport of virulence factors into host plant cells; and the development of flagella systems is closely associated with the regulation or secretion of virulence factors (Chatterjee *et al.*, 2009). Nutrient uptake from host plant tissue by plant pathogens is also a critical process in pathogenesis (Urbany & Neuhaus, 2008). There are some reports that purine and pyrimidine metabolism is related to pathogenicity (Chatterjee & Sonti, 2005; Kim *et al.*, 2003) and gluconate metabolism is required for virulence of the soft rot pathogen *P. carotovorum* subsp. *carotovorum* (Mole *et al.*, 2010).

Soft rot bacteria must attach to the plant surface and resist antibacterial chemicals produced by plants. Recently, it was reported that production of cellulose enhances plant surface colonization and is required for resistance to chlorine treatments in *Dickeya dadantii* (Prigent-Combaret *et al.*, 2012). In this bacterium, *bcs* operon is involved in the cellulose synthesis (Jahn *et al.*, 2011). However, the bacterial genes associated with attachment or survival are not well characterized in *P. carotovorum* subsp. *carotovorum*, which is a necrotroph. The interactions of necrotroph pathogens with plants are not as well understood as those of biotrophs. Previous studies have focused on PCWDE enzyme production in *P*. *carotovorum* subsp. *carotovorum* (Cui *et al.*, 2008; Laasik *et al.*, 2005), but many more factors are considered to be involved in its complex pathogenesis.

In this study, we used Tn5 transposon mutagenesis to select mutants of *P*. *carotovorum* subsp. *carotovorum* with altered ability to produce soft rot symptoms. Among a total of 5314 transposon-mediated mutants screened on Chinese cabbage, 35 mutants with reduced or no pathogenicity were isolated and 14 of those with mutations at different loci were characterized. The altered genes were associated with various functions, such as nutrient utilization, QS, motility, secretion and regulation. We report the identification of new candidate pathogenicity factors in the genus *Pectobacteria*.

## Methods

### 

#### Bacterial strains, media and cultivation conditions.

The bacterial strains and plasmids used or constructed in this study are shown in Table 1. *P. carotovorum* subsp. *carotovorum* Pcc21 (isolated in Korea) and its mutants were cultured and tested at 28 °C in LB medium (1 % tryptone, 0.5 % yeast extract, 1 % NaCl, pH 7.5). *Escherichia coli* were cultured at 37 °C. To compare mutant bacterial growth, Chinese cabbage extract, Chinese cabbage exudates and M9 salts minimal medium supplemented with various carbon sources were used. To prepare enzyme assay samples, the strains were cultured overnight in LB medium at 28 °C. When required, medium was supplemented with rifampicin, kanamycin or ampicillin at a final concentration of 50 µg ml^−1^, or with designated chemicals at the appropriate concentration.

**Table 1.** Bacterial strains and plasmids used in this study

Strain or plasmid		Relevant characteristics†		Reference or source
**Strains**				
*E. coli*				
EC100 *pir*+		F-*mcr*Δ(*mrr*-*hsdRMS*-*mcrBC*)ϕ80d*lacZ*Δ*lacX*74 *recA1 endA*1*araD*139Δ(*ara*, *leu*)7697*galU galK* γ-*rpsLnupG pir*+(DHFR)		Epicentre
*P. carotovorum* subsp. *carotovorum*				
Pcc21		WT, Rif^r^		Roh *et al.* (2010)
Pcc21-M2, 4, 5, 8~10, 13~16, 19~22		Pcc21 : : Tn5, Rif^r^, Kan^r^		This study
Pcc21-M2, 4, 5, 8~10, 13~16, 19~22-GFP	Pcc21 : : Tn5 containing pGFPuv vector in each mutant, Rif^r^, Kan^r^, Amp^r^		This study	
Plasmids*				
pNP21-2		Plasmid by self-ligation from Pcc21 Tn5 mutant, *pyrD* : : Tn5, Kan^r^		This study
pNP21-4		Plasmid by self-ligation from Pcc21 Tn5 mutant, *purH* : : Tn5, Kan^r^		This study
pNP21-20		Plasmid by self-ligation from Pcc21 Tn5 mutant, *purD* : : Tn5, Kan^r^		This study
pNP21-14		Plasmid by self-ligation from Pcc21 Tn5 mutant, *leuA* : : Tn5, Kan^r^		This study
pNP21-19		Plasmid by self-ligation from Pcc21 Tn5 mutant, *serB* : : Tn5, Kan^r^		This study
pNP21-10		Plasmid by self-ligation from Pcc21 Tn5 mutant, *expI* : : Tn5, Kan^r^		This study
pNP21-15		Plasmid by self-ligation from Pcc21 Tn5 mutant, *expR* : : Tn5, Kan^r^		This study
pNP21-5		Plasmid by self-ligation from Pcc21 Tn5 mutant, *flgA* : : Tn5, Kan^r^		This study
pNP21-9		Plasmid by self-ligation from Pcc21 Tn5 mutant, *fliA* : : Tn5, Kan^r^		This study
pNP21-21		Plasmid by self-ligation from Pcc21 Tn5 mutant, *flhB* : : Tn5, Kan^r^		This study
pNP21-22		Plasmid by self-ligation from Pcc21 Tn5 mutant, *qseC* : : Tn5, Kan^r^		This study
pNP21-8		Plasmid by self-ligation from Pcc21 Tn5 mutant, *tolC* : : Tn5, Kan^r^		This study
pNP21-16		Plasmid by self-ligation from Pcc21 Tn5 mutant, PCC21_023220 : : Tn5, Kan^r^		This study
pNP21-13		Plasmid by self-ligation from Pcc21 Tn5 mutant, ECA2640 : : Tn5, Kan^r^		This study

* Plasmid: using R6kγori in Tn5 <R6kγori/KAN-2>, we rescued the mutated region by self-ligation as a plasmid from each individual mutant.

† Rif, rifampicin; Kan, kanamycin; Amp, Ampicillin.

#### Transposon mutagenesis.

A *P. carotovorum* subsp. *carotovorum* Pcc21 mutant library was constructed using an EZ-Tn5 <R6kγori/KAN-2>Tnp Transposome™ kit (Epicentre Biotechnologies) according to the manufacturer’s protocol. Briefly, the transposome complex was introduced into *P*. *carotovorum* subsp. *carotovorum* Pcc21 by electroporation and the cells were spread on an LB agar plate supplemented with kanamycin and rifampicin. A total of 5314 single-colony-forming mutants were selected and stored at −80 °C.

#### Complementation of mutants.

To construct 14 plasmids for complementation analysis of each mutant, genes corresponding to the mutated regions of each mutant were amplified with PCR using specific primers listed in Table S1 (available in *Microbiology* Online). Analyses of the mutated genes with the database of BioCyc (Caspi *et al.*, 2010) and of OperonBD (Pertea *et al.*, 2009) predicted that the mutated genes in Pcc21-M2, Pcc21-M5, Pcc21-M8, Pcc21-M9, Pcc21-M10, Pcc21-M15 and Pcc21-M16 are monocistronic, but those in Pcc21-M4, Pcc21-M13, Pcc21-M14, Pcc21-M19, Pcc21-M20, Pcc21-M21 and Pcc21-M22 are polycistronic. In the case of polycistronic genes, the whole operon containing the mutated gene was amplified and cloned. Pcc21-M4 and Pcc21-M20 belonged to the same operon. The mutated gene in Pcc21-M22 was the last gene of the operon, and both the whole operon and the corresponding gene were amplified and cloned for further experiments (Table S2). The PCR products were purified and cloned into pGEM-T easy vector (Promega) or into pGFPuv vector (Clontech), replacing the GFP gene with one of the restriction enzyme sets (*Sph*I-*Spe*I, *Pst*I-*Apa*I or *Xba*I-*Spe*I). The constructed plasmids, pDH200-213, are listed in Table S2. The constructed plasmids were transformed into each corresponding mutant by an electroporation or conjugation method, as described previously (Lee *et al.*, 2008; Sambrook *et al.*, 1989). The *E. coli* S17-1 *λ pir* strain was used as a conjugal donor.

#### Pathogenicity test.

Fresh, woundless Chinese cabbage was rinsed twice with 70 % ethanol. All mutants were diluted and fixed at a cell density equivalent to an optical density at 600 nm (OD_600_) of 0.5. A small scar was made on the Chinese cabbage with forceps, and 10 µl of each mutant was inoculated onto the injured section. The Chinese cabbages were placed into sterilized plastic boxes with sufficient moisture and stored at 28 °C.

#### DNA sequence determination and analysis.

Mutant genomic DNA was digested with *Eco*RI or *Kpn*I, which did not have recognition sites within the transposon. Fragmented genomic DNA was self-ligated and used to transform *E. coli* EC100 *pir*+ by the CaCl_2_ method (Sambrook *et al.*, 1989). Plasmids containing the mutated region were isolated using a DNA-spin plasmid DNA isolation kit (Intron Biotechnology), and the inserts were sequenced using the specific primers KAN-2 FP-1 and R6KAN-2 RP-1 (Epicentre). To identify nucleotide sequences similar to the mutated regions, we searched the GenBank database using blast (http://www.ncbi.nlm.nih.gov/).

#### Mutant growth in Chinese cabbage extract and exudate.

To prepare disinfected Chinese cabbage extract, Chinese cabbage was ground using a mixer. Debris was removed by centrifugation at 5800 ***g*** for 30 min, and the supernatant was collected. This step was performed at least five times. Finally, the collected supernatant was sterilized using a Stericup (0.22 µm pore size, GP Express Plus Membrane, Millipore). Chinese cabbage exudate was prepared according to the following method. White leaf regions were rinsed with water and cut uniformly. Chinese cabbage exudate was collected by centrifugation at 3300 ***g*** for 5 min and filtered through filter paper to remove debris. The filtrate was sterilized by passing through a 0.22 µm pore CA membrane filter (Corning), which eliminated small debris and bacteria. Sterilized Chinese cabbage extract or exudate containing appropriate antibiotics was inoculated with each mutant, and growth was measured with a Lambda 25 UV/VIS spectrometer (PerkinElmer).

#### *In vivo* multiplication analysis.

To track the mutant multiplication path on Chinese cabbage, we introduced pGFPuv plasmid (Clontech) into the WT Pcc21 and into each mutant, using the CaCl_2_ method (Sambrook *et al.*, 1989). These strains were inoculated onto Chinese cabbage using the same method as applied in the pathogenicity test. GFP expression was measured every 12 h using a luminescent image analyser (JP/LAS-3000, FujiFilm).

#### Extracellular enzyme assays.

Bacterial cells were removed from overnight cultures and the supernatants were used for enzyme assays. The compositions of the media for the semiquantitative enzyme activity assays were as follows: Pel assay medium contained 1 % polygalacturonic acid (PGA), 1 % yeast extract, 0.38 µM CaCl_2_ and 100 mM Tris/HCl, pH 8.5; Peh assay medium contained 1 % PGA, 1 % yeast extract, 2.2 mM EDTA and 110 mM sodium acetate, pH 5.5; Cel assay medium contained 1 % carboxymethyl cellulose and 25 mM sodium phosphate, pH 7.0; and Prt assay medium contained 1 % skim milk and 0.1 % yeast extract (Chatterjee *et al.*, 1995; Wery *et al.*, 2003). All assay media were supplemented with 0.8 % agarose and 0.2 % sodium azide. In each plate, wells were made using a No. 2 cork borer, and the bottoms of the wells were covered with molten 0.8 % agarose containing 0.2 % sodium azide. Overnight cultures grown in LB medium were centrifuged at 5800 ***g*** for 5 min, and 30 µl of the supernatant was added to each well. After incubation at 28 °C for 16–18 h, 4 N HCl was poured onto the Peh and Pel enzyme assay plates, and the clear zones were measured. The Cel assay plates were developed with 0.1 % Congo red solution for 15–30 min and washed several times with 1 M NaCl until clear zones were visible around the holes. The Prt assay plates were measured after 36 h of incubation, revealing clear zones without any further treatment.

Pel activity was measured as described by Starr *et al.* (1977) with some modifications. Briefly, 10 µl supernatant was mixed with 990 µl Pel reaction mixture (0.1 M Tris/HCl, pH 8.5, 2.2 mM CaCl_2_ and 5.75 mg ml^−1^ PGA) and then incubated at room temperature. Absorbance changes were measured at 235 nm for 10 min using a Lambda 25 UV/VIS spectrometer (PerkinElmer). The activity was calculated as A_235 nm_ h^−1^ OD_600_ nm^−1^ and a relative ratio of each mutant versus WT strain was calculated.

#### Motility test on agar plates and transmission electron microscopy.

Motility tests were performed on semi-solid LB medium (0.3 % agar). The plates were inoculated with bacteria by stabbing and incubated at 28 °C. Motility was compared after 12 and 24 h of incubation (Sperandio *et al.*, 2002).

For transmission electron microscopy, cells freshly grown on Tryptic Soy Agar plates were resuspended in sterile water. Cells were negatively stained with 0.1 % (w/v) uranyl acetate and observed with a transmission electron microscope (Carl Zeiss LEO 912 AB).

#### Quantitative biofilm formation assessment.

To assess biofilm formation, cells were cultured overnight at 28 °C in LB and then diluted with fresh LB medium to a cell density equivalent to an OD_600_ of 0.2–0.3 (~10^8^ c.f.u. ml^−1^). The wells of 96-well flat-bottom polystyrene microplates (SPL) were filled with 200 µl of diluted cells per well, followed by incubation for 48 h at 28 °C in the static state. The cultured cells were removed, and the wells were rinsed with PBS. The biofilm was stained with 1 % crystal violet solution for 20 min, the wells were washed twice with deionized water and the samples were eluted with 200 µl of 95 % ethanol. To quantify the amount of biofilm, the absorbance of 100 µl of each 200 µl sample was measured at 570 nm using a microplate reader.

#### Antimicrobial chemical susceptibility test.

Changes in antimicrobial susceptibility were evaluated using a MIC assay (Al-Karablieh *et al.*, 2009a; Barabote *et al.*, 2003). The following plant-derived chemicals were purchased from Sigma-Aldrich: berberine, *t*-cinnamic acid, *p*-coumaric acid, esculetin, genistein, gossypol, plumbagin, pyrithione and rhein. Stock solutions were made by dissolving each chemical in deionized water or an appropriate solvent: water for berberine and pyrithione, 100 % methanol for esculetin, 50 mM NaOH for genistein and rhein, and 95 % ethanol for the others. Overnight cultures of strains were diluted with fresh LB medium to a cell density of 10^6^ cells ml^−1^, and 100 µl of the cells were added to the wells of 96-well flat-bottom polystyrene microplates. 100 µl of twofold serially diluted antimicrobial chemicals were added to each well. Serially diluted solvents were included as controls for solvent side effects. The microplates were incubated at 28 °C with shaking at 170 r.p.m. for 24 h. The MIC was determined as the minimum concentration of a chemical that prevented visible growth.

## Results and Discussion

### Isolation of pathogenicity-defective mutants and identification of mutated regions

To isolate novel pathogenicity-related genes, we used a simple mutagenesis system based on a transposon–transposase synaptic complex (transposome). Tn5 transposons contain an R6Kγ conditional origin of replication, and the Tn903 kanamycin resistance gene was employed as a random mutational element. After screening 5314 mutants, we isolated 35 mutants that showed reduced or no pathogenicity in Chinese cabbage.

The transposon insertion sites from the 35 clones were sequenced, and a blast search of the GenBank database showed that the 35 mutants carried Tn5 transposons in 14 different loci (Table 2). Pathogenicity-related loci identified in the *P*. *carotovorum* subsp. *carotovorum* genome were predicted to encode proteins involved in various functions, such as nutrient utilization, QS, motility, secretion and regulation. Of the 35 mutants, 14 typical mutants representing individual genes were selected and we carried out complementation tests to confirm the ORFs of the genes. We introduced constructed clones into corresponding mutants and then a pathogenicity test was performed (Table S2). The pathogenicity against Chinese cabbage of all mutants except Pcc21-M4 and Pcc21-M20 containing the corresponding WT gene or operon was successfully restored (data not shown). Introduction of pDH201 into Pcc21-M4 and Pcc21-M20, representing *purH* and *purD* genes, in one operon failed and the reasons remain unclear.

**Table 2.** Genes mutated in the mutants showing reduced or no pathogenicity

Mutant	Homologous gene	Predicted function*	Identity†	
%	Origin of the gene
Pcc21-M2	*pyrD*	Dihydroorotate dehydrogenase	100	PBR1692
Pcc21-M4	*purH*	Bifunctional phosphoribosylaminoimidazole carboxamide formyltransferase/IMP cyclohydrolase	100	PBR1692
Pcc21-M20	*purD*	Phosphoribosylamine-glycine ligase	99	PBR1692
Pcc21-M14	*leuA*	2-Isopropylmalate synthase	99	PBR1692
Pcc21-M19	*serB*	Phosphoserine phosphatase	99	PBR1692
Pcc21-M10	*expI*	Synthesis of *N*-(3-oxohexanoyl)-l-homoserine lactone	99	WPP14
Pcc21-M15	*expR*	Quorum-sensing transcriptional regulator	98	Ecc71
Pcc21-M5	*flgA*	Flagellar basal body P-ring biosynthesis protein	98	PBR1692
Pcc21-M9	*fliA*	Flagellar biosynthesis sigma factor	99	WPP163
Pcc21-M21	*flhB*	Flagellar biosynthesis protein	98	WPP14
Pcc21-M22	*qseC*	Sensor protein QseC bacterial adrenergic receptor	96	WPP14
Pcc21-M8	*tolC*	Outer membrane channel protein	99	PC1
Pcc21-M16	PCC21_023220	Putative DNA-binding protein	98	PBR1692
Pcc21-M13	ECA2640‡	Unknown	99	WPP14

* Gene information was retrieved from *P. carotovorum* subsp. *carotovorum* Pcc21 complete genome data (GenBank accession no. NC_018525.1).

† Protein homology analysis of mutated genes was performed using the blastp program (NCBI/blast) and the highest homologous gene information is listed. Identity values are listed as percentages. Origin strain of homologous genes was retrieved from one of the following strains: *P. carotovorum* subsp. *carotovorum* Ecc71, *P. carotovorum* subsp. *brasiliensis* PBR1692, *P. carotovorum* subsp. *carotovorum* PC1, *P. carotovorum* subsp. *carotovorum* WPP14, *P. wasabiae* WPP163.

‡ ECA2640 was retrieved from the *P. atrosepticum* SCRI1043 genome sequence (GenBank accession no. NC_004547.2).

### Bacterial multiplication in Chinese cabbage

**Fig. 1.**
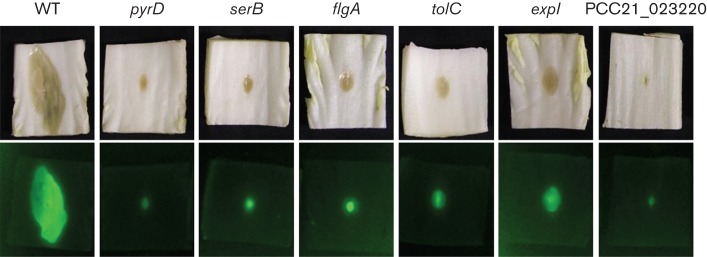
Strain multiplication in Chinese cabbage. WT Pcc21 and its mutants harbouring the pGFPuv plasmid were separately inoculated on an injured section of Chinese cabbage. After an appropriate incubation time, GFP expression was visualized using a luminescent image analyser. WT, *P. carotovorum* subsp. *carotovorum* Pcc21. These pictures are representative of three independent experiments.

Most mutants did not induce soft rot symptoms in Chinese cabbage. However, strains with mutations in *flhB*, *qseC* or *tolC* homologous genes showed soft rot symptoms 24–48 h later than the other mutants (Table 3). We tested whether bacterial growth is a requirement for visual soft rot symptoms or not by monitoring bacterial growth with bacteria carrying the GFP gene. Green fluorescence was observed only around areas showing soft rot symptoms, but not observed beyond those symptoms. Thus, it appears that when the bacteria multiply, soft rot symptoms occur at the same time (Fig. 1). All mutants multiplied under adequate nutritional conditions (such as in the presence of plant extracts), but limited nutrient availability (such as in the presence of plant exudates) inhibited the growth of several mutants, particularly those carrying mutations in the *pyrD* and *purH* homologous region (Table 3).

**Table 3.** Growth of WT Pcc21 and its mutants in various media, and pathogenicity test results in Chinese cabbage

Strain	Homologous gene	Pathogenicity test*				Growth in Chinese cabbage†		M9 MM+0.4 % carbon source‡
12 h	24 h	48 h	84 h	Extract	Exudate
Pcc21§	WT	+	++	+++	+++	+++	+++	+
Pcc21-M2	*pyrD*	–	–	–	–	+++	+	–
Pcc21-M4	*purH*	–	–	–	–	+++	+	–
Pcc21-M20	*purD*	–	–	–	–	+++	++	–
Pcc21-M14	*leuA*	–	–	–	–	+++	++	–
Pcc21-M19	*serB*	–	–	–	–	++	++	–
Pcc21-M10	*expI*	–	–	–	–	+++	++	+
Pcc21-M15	*expR*	–	–	–	–	+++	++	+
Pcc21-M5	*flgA*	–	–	–	–	+++	+++	+
Pcc21-M9	*fliA*	–	–	–	–	+++	+++	+
Pcc21-M21	*flhB*	–	–	+	+++	+++	+++	+
Pcc21-M22	*qseC*	–	–	+	++	+++	++	+
Pcc21-M8	*tolC*	–	–	+	+	+++	+++	+
Pcc21-M16	PCC21_023220	–	–	–	–	+++	+++	+
Pcc21-M13	ECA2640||	–	–	–	–	+++	++	+

* Rotten zone diameters were measured and scored as: −, <0.3 cm; +, 0.3–0.6 cm; ++, 0.6–0.9 cm; and +++, >0.9 cm. The data are representative of three independent experiments.

† Growth of mutants was compared with WT Pcc21 growth after inoculation for 24 h and scored as: +++, similar to WT; ++, 20–50 % reduction was observed; +, >50 % reduction. The average growth ratio was obtained from at least two independent experiments.

‡ Glycerol, sucrose, fructose, glucose and mannitol were used as sole carbon sources. All five carbon sources showed the same growth results. +, grew well; –, no growth. The data are representative of three independent experiments.

§ Pcc21, *P. carotovorum* subsp. *carotovorum* Pcc21.

||ECA2640 was retrieved from the *P. atrosepticum* SCRI1043 genome sequence (GenBank accession no. NC_004547.2).

### Mutants with altered nutrient utilization

The ability of the mutants to use a carbon source was investigated by cultivating the mutants in M9 minimal medium with the separate addition of glycerol, sucrose, fructose, glucose and mannitol. Strains carrying mutations at five different genes, Pcc21-M2, Pcc21-M4, Pcc21-M14, Pcc21-M19 and Pcc21-M20, did not grow on minimal medium with all five different carbon sources (Table 3). Sequence analysis of five mutated genes revealed that they are associated with nutrient utilization. Pcc21-M2 had transposon insertion in a class 2 dihydroorotate dehydrogenase homologous region, which is related to pyrimidine synthesis (Table 2) (Aleksenko *et al.*, 1999; West, 2005). Mutants Pcc21-M4 and Pcc21-M20 had transposon insertions in two purine biosynthesis protein genes, bifunctional phosphoribosylaminoimidazole carboxamide formyltransferase/IMP cyclohydrolase and phosphoribosylglycinamine-glycine lygase, respectively (Chatterjee & Sonti, 2005; Dougherty *et al.*, 2006). Pcc21-M14 and Pcc21-M19 had mutations in 2-isopropylmalate synthase and phosphoserine phosphatase, which are involved in the biosynthesis of leucine and serine, respectively (Table 2) (Patte *et al.*, 1999; Wang *et al.*, 2007; Zou *et al.*, 2007). These results suggest that the virulence deficiency of mutants with insertions at five different loci is due to an inability to produce pyrimidine, purine, leucine or serine.

*P. carotovorum* subsp. *carotovorum* replicates in the intercellular space until conditions favour disease development. It is not known whether non-pathogenic mutants continue to multiply in vascular tissue or not. The 14 mutants carrying GFP did not multiply efficiently in the Chinese cabbage intercellular space as expanded GFP expression was not observed in Chinese cabbage (Fig. 1). However, all mutants (excluding the five mutants carrying transposon insertions in nutrient utilization-related genes) multiplied in Chinese cabbage exudates (Table 3), suggesting that other factors, as well as nutrient utilization, may be involved in the pathogenicity-defective mutants.

### Mutants with altered production and secretion of exo-enzymes

**Fig. 2.**
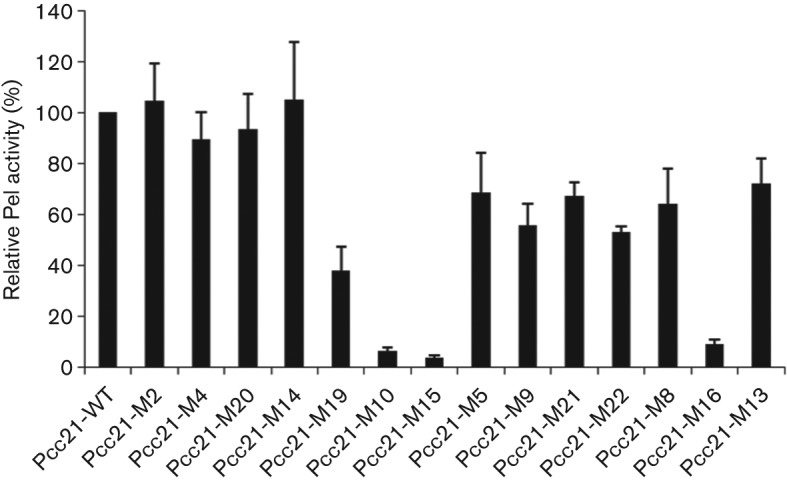
Quantitative Pel activity of WT Pcc21 and its mutants. Supernatants of overnight cultures were mixed with the reaction mixture and then incubated at room temperature, as described in Methods. The values are the means and standard deviations of triplicate experiments.

To determine whether the loss in pathogenicity in specific mutants is caused by reduced production or secretion of exo-enzymes, the 14 mutants were tested for the production and secretion of four different PCWDEs. Only three of the 14 did not produce or secrete exo-enzymes (Table 4 and Fig. 2). These mutants lost over 90 % of Pel activity, which is a major PCWDE of plant pathogen bacteria, compared with the WT strain. Two mutants carried transposon insertions in the QS-associated genes, *expI* and *expR*, and the other had an insertion in the PCC21_023220 gene with an unknown function. The disruption of *expI* or *expR* affected the production or secretion of exo-enzymes because exo-enzyme production and secretion are controlled by a QS signal produced when the bacteria reach a specific cell density. The disrupted gene in Pcc21-M10 (*expI*) is involved in the synthesis of AHL, which is a major QS signal. We also isolated strains with mutations in *expR* (Pcc21-M15), which is known as a AHL receptor, the transcription regulator of *rsmA* (rsm, repressor of secondary metabolites) (Table 2) (Burr *et al.*, 2006; Chatterjee *et al.*, 1995; Cui *et al.*, 2006; Fray *et al.*, 1999). Pcc21-M16 had a transposon insertion at the locus PCC21_023220. The PCC21_023220 gene encodes a putative DNA-binding protein with a YheO-like PAS (per-arnt-sim) domain. PAS domains are found in a variety of prokaryotes and eukaryotes as important signalling modules (Gu *et al.*, 2000; Taylor & Zhulin, 1999). These findings suggest that PCC21_023220 may be involved in the production or secretion of exo-enzymes through unknown pathways as a regulatory protein (Table 4). Many bacteria carry this gene, but its function has not been determined. This is the first report that PCC21_023220 controls the production or secretion of PCWDE.

**Table 4.** Summary of mutant phenotypes, including altered PCWDE production, motility and biofilm formation

Strain	Homologous gene	PCWDE production (mm)*				Motility	Biofilm formation (%)†
Pel	Peh	Prt	Cel
Pcc21‡	WT	5.8±0.6	5.2±1.0	5.9±0.1	5.8±0.2	+	100
Pcc21-M2	*pyrD*	7.3±0.3	4.2±0.3	5.3±0.2	6.1±0.4	+	90±17.1
Pcc21-M4	*purH*	7.6±0.6	5.5±0.6	6.6±0.3	6.0±0.3	+	70±16.5
Pcc21-M20	*purD*	6.1±0.6	4.1±0.2	6.5±0.4	6.8±0.4	+	71±22.0
Pcc21-M14	*leuA*	7.6±0.6	4.8±0.3	5.6±0.6	6.9±0.1	+	68±10.8
Pcc21-M19	*serB*	5.5±0.7	3.9±0.4	5.1±0.1	6.8±0.4	+	66±1.6
Pcc21-M10	*expI*	0.0	0.0	0.0	0.0	+	23±6.3
Pcc21-M15	*expR*	0.0	0.0	0.0	0.0	+	22±6.3
Pcc21-M5	*flgA*	6.2±0.6	4.1±0.4	5.7±0.4	6.5±0.7	–	38±21.6
Pcc21-M9	*fliA*	6.2±0.6	3.6±0.4	5.4±0.6	5.5±0.7	–	39±17.4
Pcc21-M21	*flhB*	6.8±0.3	3.5±0.4	2.9±0.2	6.4±0.1	–	50±28.0
Pcc21-M22	*qseC*	7.4±0.3	3.9±0.3	6.2±0.2	6.3±0.4	+	27±7.7
Pcc21-M8	*tolC*	4.5±0.5	3.1±0.1	3.2±0.3	6.0±0.4	+	125±27.1
Pcc21-M16	PCC21_023220	0.0	0.0	0.0	0.0	+	87±18.1
Pcc21-M13	ECA2640§	6.2±0.6	4.2±0.3	6.4±0.3	7.3±0.4	+	60±35.0

* Enzyme activities were measured as the length of clear haloes from inoculated holes. The data represent the average values and standard deviations of at least three independent experiments.

† Average values after 48 h of incubation. Average values and standard deviations were obtained from at least three independent experiments.

‡ Pcc21, *P. carotovorum* subsp. *carotovorum* Pcc21.

§ ECA2640 was retrieved from the *P. atrosepticum* SCRI1043 genome sequence (GenBank accession no. NC_004547.2).

It is interesting to note that *P. carotovorum* subsp. *carotovorum* Pcc21 has more than eight pectate lyases, three polygalacturonases, two cellulases and several putative PCWDE-associated genes (GenBank accession no. NC_018525.1). An insertional mutation in just one of these genes was not sufficient to cause deficient pathogenicity. None of our mutants had insertions in the gene encoding PCWDE.

### Mutants with altered motility

**Fig. 3.**
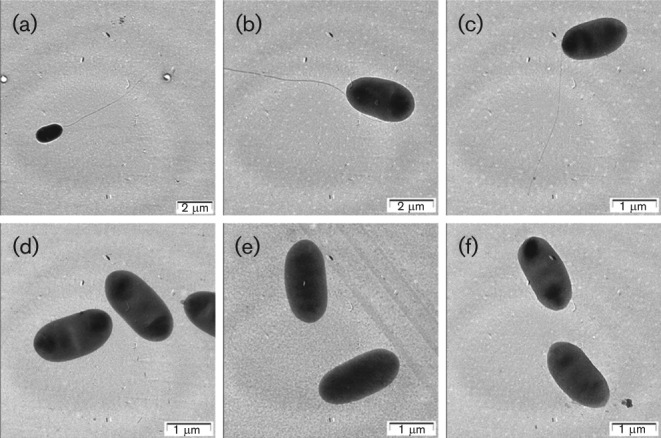
Transmission electron microscopy of WT Pcc21 and its mutants. Cells were negatively stained with 0.1 % (w/v) uranyl acetate. (a) and (b), WT Pcc21; (c), Pcc21-M22 (*qseC*); (d), Pcc21-M5 (*flgA*); (e), Pcc21-M9 (*fliA*); (f), Pcc21-M21 (*flhB*).

Motility is an important determinant for the pathogenesis of *Pectobacteria* (Cui *et al.*, 2008; Hossain *et al.*, 2005). We investigated the motility of all 14 mutants on semi-solid agar plates. All mutants, except Pcc21-M5, Pcc21-M9 and Pcc21–M21, were motile. Mapping of the transposon insertions of non-motile mutants showed insertions in *flgA*, *fliA* and *flhB*, which code for proteins involved in flagella biosynthesis (Ferris *et al.*, 2005; Kutsukake *et al.*, 1990; Sperandio *et al.*, 2002). Pcc21-M5 had a mutation in the *flgA* homologous region, which codes for the flagellar basal body P-ring. Pcc21-M9 had a mutation in the *fliA* homologous region, which encodes a sigma factor for flagella synthesis, and Pcc21-M21 had a mutation in *flhB*, which encodes a flagella biosynthesis protein (Table 4). In Pcc21-M22, a transposon was located in a *qseC* homologous gene, which encodes the sensor kinase in a two-component regulatory system in *E. coli* (EHEC) and *Salmonella* Typhimurium (Clarke *et al.*, 2006; Hadjifrangiskou *et al.*, 2011). In the absence of QseC, QseB is constitutively phosphorylated and represses virulence-associated genes, including those for type 1 pili, curli and flagella (Hadjifrangiskou *et al.*, 2011). However, Pcc21-M22 has a movement radius nearly equal to that of WT Pcc21. Transmission electron microscopy of these mutants revealed that Pcc21-M5, Pcc21-M9 and Pcc21-M21 had no flagella, while Pcc21-M22 retained flagella (Fig. 3).

### Mutants with altered biofilm formation

Biofilm formation is crucial for the pathogenesis of plant and animal bacterial pathogens. We evaluated the biofilm formation of mutants using the crystal violet staining assay, which has been used to assess biofilm formation by a wide variety of bacteria (Koczan *et al.*, 2009; O’Toole & Kolter, 1998; Pérez-Mendoza *et al.*, 2011; Wakimoto *et al.*, 2004). After growing for 48 h in a 96-well assay plate, *P. carotovorum* subsp. *carotovorum* Pcc21 demonstrated visual evidence of biofilm formation, whereas there was an absence of biofilm formation by Pcc21-M10 (*expI*), Pcc21-M15 (*expR*) and Pcc21-M22 (*qseC*). Next, we quantified the amount of biofilm formation. The amount of biofilm formed by the Pcc21-M22 (*qseC*) mutant was only 20 % of the amount formed by the WT bacteria (Table 4). This result supports recent findings that *qseC* is involved in biofilm formation (Durham-Colleran *et al.*, 2010; González Barrios *et al.*, 2006; Novak *et al.*, 2010). The mutation in the *qseC* gene also reduced pathogenicity. Interestingly, biofilm formation by *P. carotovorum* subsp. *carotovorum* Pcc21 is perhaps controlled by two different QS signal pathway systems; one is *expI* and *expR*, and the other is *qseC* and PCC21_042560. However, the QS signal system genes *expI* and *expR* appeared to control both PCWDE and biofilm production, and mutations in these genes abrogated pathogenicity. Three mutants with defective flagella biosynthesis exhibited reduced biofilm formation. The *flgA* and *fliA* mutants produced less than 50 % of the biofilm produced by WT *P. carotovorum* subsp. *carotovorum* Pcc21. These results suggest that flagella play a role in biofilm formation in *P. carotovorum* subsp. *Carotovorum*, as in *E. coli.* It has been known that motility is important for normal biofilm formation in *E. coli* (Pratt & Kolter, 1998).

### Mutants with different resistance to antibacterial plant chemicals

Plants produce a variety of chemicals with antimicrobial properties (Federici *et al.*, 2004; Simões *et al.*, 2009), and many bacteria have evolved with mechanisms that tolerate or eliminate plant-derived toxins (Federici *et al.*, 2004). Given that none of the mutants grew efficiently in Chinese cabbage, the effects of plant-derived chemicals on bacterial growth were evaluated. Of the mutants, only the *tolC* mutant showed increased sensitivity to half of the plant-derived chemicals that were evaluated (Table 5). The MICs of berberine, rhein and genistein in the *tolC* mutant strain were at least 128-, 64- and 64-fold lower, respectively, than the MICs in WT bacteria (Table 5). Rhein is an antimicrobial of rhubarb. Genistein is an isoflavonoid produced as a precursor in the biosynthesis of antimicrobial pytoalexins and phytoanticipins. The alkaloid berberine is a common component of several plant species, particularly in the Berberidaceae family. Although berberine has antibiotic properties, it is exported by bacterial multidrug resistance pumps. The susceptibility of the *tolC* mutant to berberine increased 128-fold, while susceptibility to phenolic compounds, such as coumaric acid, cinnamic acid and pyrithione, was not affected. Different susceptibilities to diverse phytochemicals suggest that TolC may play a role in antibacterial resistance via efflux systems. Recently, several researchers suggested a relationship between TolC and a multidrug resistance efflux pump in other plant-pathogenic bacteria (Al-Karablieh *et al.*, 2009b; Barabote *et al.*, 2003). Considering that Pcc21-M8 (*tolC*) showed decreased exo-enzyme production in some tests rather than that of the WT strain, the type I secretion system and the type II secretion system are likely to be involved in *P. carotovorum* subsp. *carotovorum* infection of host cells.

**Table 5.** Susceptibility of WT Pcc21 and other mutants to plant-derived chemicals

Plant-derived chemical	Strain [MIC (μg ml^−1^)]*			
Pcc21† WT	Pcc21-M2‡ *pyrD*	Pcc21-M8 *tolC*	Several fold difference§
Berberine	2000	2000	15.63	128
*t*-Cinnamic acid	1000	1000	1000	
*p*-Coumaric acid	500	500	500	
Esculetin	500	500	250	2
Genistein	1000	1000	15.63	64
Gossypol	>500	>500	>500	
Plumbagin	62.5	62.5	15.63	4
Pyrithione	15.63	15.63	15.63	
Rhein	>1000	>1000	15.63	>64

* Owing to solubility limitations, some plant chemicals could not be tested at higher concentrations. The data are representative of three independent experiments.

† Pcc21, *P. carotovorum* subsp. *carotovorum* Pcc21.

‡ The other mutants showed susceptibility values similar to that of Pcc21-M2.

§ Fold increase represents the ratio of the susceptibility to antimicrobial plant chemicals compared with WT Pcc21.

The Pcc21-M13 mutant showed PCWDE production comparable to the WT and retained motility, but produced about 60 % of biofilm compared to the WT (Table 4). Pcc21-M13 has a transposon insertion in the ECA2640 homologous gene and the function of this uncharacterized gene in pathogenicity remains to be studied.

In summary, we identified three novel genes associated with the pathogenicity of *P. carotovorum* subsp. *carotovorum*: *qseC*, *tolC* and PCC21_023220. The function of PCC21_023220 as a regulator remains unknown. We are currently investigating the role of PCC21_023220 as a regulator of PCWDE production. Although many genes that function in *P. carotovorum* subsp. *carotovorum* pathogenicity have been identified, many more uncharacterized genes important for pathogenesis are waiting to be found.
